# Proteomic Analysis of the Alterations in Follicular Fluid Proteins During Oocyte Maturation in Humans

**DOI:** 10.3389/fendo.2021.830691

**Published:** 2022-02-03

**Authors:** Chong Wang, Xiaoyang Fei, Hongyan Zhang, Wenjing Zhou, Zhaojun Cheng, Ying Feng

**Affiliations:** ^1^ Reproductive Medicine Center, Hangzhou Women’s Hospital (Hangzhou Maternity and Child Health Care Hospital), Hangzhou, China; ^2^ Affiliated Hangzhou First People’s Hospital, Zhejiang University School of Medicine, Hangzhou, China

**Keywords:** follicular fluid, proteomic, iTRAQ, oocyte maturation, diminished ovarian response (DOR)

## Abstract

Many components in ovarian follicles (follicular fluid, cumulus cells, granular cells, etc.) dynamically change during folliculogenesis and play a positive or negative role in oocyte maturation. Infertile women who underwent intracytoplasmic sperm injection (ICSI) treatment in the reproductive medicine centre of Hangzhou Women’s Hospital between October 2018 and October 2021 were included. The ovarian follicular fluid and cumulus cells of diminished ovarian response (DOR) patients and control subjects with medical records of clinical data were collected. In total, 31 differentially expressed proteins, including 10 upregulated proteins (>1.50-fold, *P*<0.05) and 21 downregulated proteins (<0.67-fold, *P*<0.05), were identified in mature *vs.* immature oocytes by iTRAQ labelling coupled with 2D LC-MS/MS. GO analysis revealed that ‘cell population proliferation’ was the most diverse enrichment trend between up/downregulated proteins, while phagosome process and the PI3K-Akt signaling pathway were the two most significant pathways revealed by KEGG enrichment classification. Human prostatic acid phosphatase (PAP, ACPP) and CD5 antigen-like (CD5L) were two proteins verified by ELISA to be differentially expressed between MII and Gv oocytes (*P*<0.0001 and *P*<0.0001, respectively). Further measurement found significantly lower level of ACPP in follicular fluids and cumulus cells of DOR patients (*P*=0.028 and *P*=0.004, respectively), as an indicator of oocyte quality. Otherwise, CD5L level is upregulated in follicular fluid of DOR patients (*P*<0.0001). Our study provided experimental data to establish the objective indicator of oocyte maturation in the microenvironment of ovarian follicles, and also provided new insight into the measurement of oocyte quality.

## Introduction

Infertility is the third most common disease after tumours and cardiovascular disease. According to a population-based cross-sectional study, one in four couples of childbearing age in China suffers from infertility (1). With the increase in reproductive age due to delayed childbearing, the number of patients with diminished ovarian response (DOR) is increasing in China, resulting in non-ideal mature oocyte numbers and inadequate oocyte quality by normal exogenous gonadotropin therapy (2).

Folliculogenesis is a complex network of interacting cellular signals between somatic cells and oocytes (3). Well-regulated folliculogenesis is crucial for generating developmentally competent oocytes for fertilization. Many components in ovarian follicular fluid (e.g., proteins, cell growth factors, peptide hormones, steroids, energy metabolites) dynamically change with the growth and development of oocytes and play a positive or negative role in oocyte maturation (4). It has been reported that the levels of estrogen (5), melatonin (6), and soluble receptor for advanced glycation end-products (7) in ovarian follicular fluid can reflect the maturity level of oocytes. Previous proteomic research highlighted midkine as a crucial protein involved in folliculogenesis by studying follicular fluid from human small antral follicles (8). WAP four-disulfide core domain protein 2 was also validated by Liu et al. as a potentially follicular fluid biomarker for the diagnosis of oocyte maturation arrest caused by overweight status (9). To date, the oocyte maturation mechanism remains unclear.

This study investigated the relationship between differentially expressed proteins in human ovarian follicular fluid that represent oocyte maturity by iTRAQ labelling coupled with 2D LC-MS/MS. We characterized and analyzed differentially expressed proteins from the ovarian follicular fluid of women with different oocyte maturities (metaphase II and germinal vesicle stage) and explored the possible changes in diminished ovarian response (DOR) patients, providing support for further functional studies on validated proteins.

## Materials and Methods

### Ethical Statement

This study was approved by the Ethics Committee of the Faculty (Hangzhou First People’s Hospital, approval number: 2017–479, 2020–045–01). Written informed consent was obtained from all subjects before collection.

### Sample Collection

**Figure 1:**
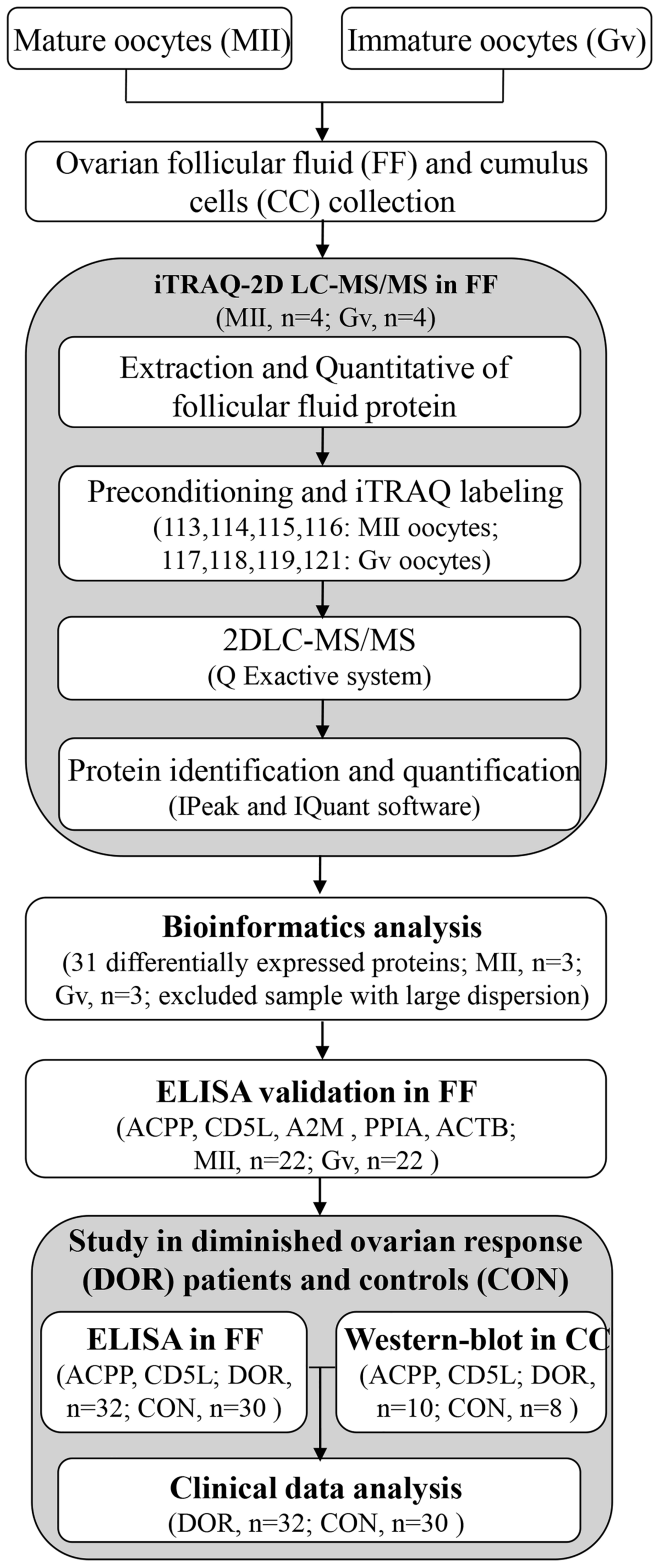
The workflow for ovarian follicular fluid biomarkers of maturation and quality of oocytes.

The whole workflow is described in **Figure 1**. We recruited a total of 22 patients undergoing intracytoplasmic sperm injection (ICSI) with both mature and immature oocytes. Ovarian follicular fluid was collected from each follicle separately and allocated according to the maturity of degranulated oocytes. Furthermore, we recruited a total of 62 patients and allocated them to two groups: the control group (n=30) and the DOR group (n=32). Antral follicle count (AFC) and anti-Mullerian hormone (AMH) were two main parameters used to classify DOR. Patients with poor ovarian reserve prestimulation parameters (AFC <7, AMH <1.2 ng/mL) were defined as having DOR per the Poseidon and Bologna criteria (10, 11). This study was conducted from October 2018 to October 2021. All the procedures were carried out in conformity with the Declaration of Helsinki.

Ovarian follicular fluid was collected by transvaginal ultrasound-guided puncture. Immediately following oocyte retrieval, follicular fluid was collected and then centrifuged at 3000 g for 15 min to collect the supernatant. Cumulus cells were collected from cumulus-oocyte complexes at the same time. The supernatant and cumulus cells were frozen at -80°C and stored until protein extraction.

### Follicular Fluid Protein Extraction

Low-abundance proteins were enriched by using the Protein Enrichment Small-Capacity Kit (1633006, Bio-Rad, USA). The protein was dissolved in lysis buffer (8 M urea, 4% CHAPS, 40 mM Tris-HCl, 1 mM PMSF 10 mM DTT), and the protein concentration was determined by the Bradford assay. Protein digestion was performed by means of the FASP method with trypsin (Promega, Madison, WI) and 0.1 M TEAB as buffer solution (12). Four individual biological replicates were pooled for iTRAQ analysis.

### Protein Digestion and iTRAQ-2D LC-MS/MS

A total of 100 μg protein from each group was digested using trypsin (Promega, USA) at a 1:50 trypsin-to-protein mass ratio at 37°C for 16 h. Then, peptides were reconstituted in 0.2 M TEAB and processed according to the manufacturer’s protocol for 8-plex iTRAQ reagent (AB SCIEX, Framingham, MA, USA). Four biological replicates of the MII group were labelled with 113, 114, 115 and 116 isobaric tags. The peptides with four biological replicates of the Gv group were labelled with 117, 118, 119 and 121 isobaric tags.

The peptide mixtures were subjected to a first-dimensional fractionation procedure using a high pH reversed-phase chromatography column (Phenomenex, Gemini-NX 3u C18110A, 150*2.00 mm). A total of 16 fractions were finally collected and dried for the subsequent LC-MS analysis. The lyophilized peptide fractions were resuspended in 2% acetonitrile containing 0.1% formic acid and loaded into a C18 trap column (Acclaim PepMap 75 μm × 150 mm, C18, 3 μm, 100 A). Online chromatographic separation was performed on a nanoLC system (Dionex Ultimate 3000 RSLCnano). The trapping and desalting procedures were carried out at a flow rate of 3 μL/min for 5 min with 100% solvent A (0.1% formic acid, 2% acetonitrile and 98% water). Then, peptides were eluted using a 65-min gradient of buffer A (0.1% formic acid) to buffer B (80% ACN containing 0.1% formic acid) at 300 nL/min. It was used on an analytical column (Acclaim PepMap 75 μm × 15 cm C18-CL, 3 μm 100 Å, Thermo160321). The information-dependent acquisition (IDA) mass spectrum technique was used to acquire tandem MS data on a Q Exactive system (Thermo Scientific) fitted with a Nanospray ion source. Data were acquired using an ion spray voltage of 2.2 kV. MS spectra were acquired across the scan range of 350–1800 m/z at a resolution of 70,000 using the maximum injection time (60 ms) per spectrum. The twenty most intense precursors per MS cycle were selected for fragmentation detected with a 100-ms maximum injection time. Tandem mass spectra were recorded at a resolution of 17,500 with the rolling collision energy on and the iTRAQ reagent collision energy adjustment on. For accurate mass measurements, the lock mass option was enabled. Dynamic exclusion was set for 10 s.

The MS/MS data were analyzed using IPeak and IQuant software to obtain protein identification and quantification (13, 14) and the NCBI *human* genome. Principal component analysis (PCA) and Pearson’s correlation coefficient were used to assess the quality of the quantitative results. Only proteins identified at global FDR ≤ 1% with ≥ 1 peptide were considered for protein lists and further downstream analysis. To confirm a differentially expressed protein, the protein was required to be identified and quantified with at least 1 significant peptide, the *P*-values for protein quantitation were less than 0.05, and the fold change was ≥ 1.5.

### Bioinformatics Analysis

First, the expression of the identified proteins was analyzed to screen out the differentially expressed proteins. All the identified proteins were functionally annotated and classified by Gene Ontology (GO, http://www.geneontology.org), Kyoto Encyclopedia of Genes and Genomes (KEGG, http://www.genome.jp/kegg/ or http://www.kegg.jp/), COG (clusters of orthologous groups of proteins), KOG (clusters of protein homology) and other databases. We performed GO, KEGG, COG, KOG and clustering analyses on all differentially expressed proteins. The protein–protein network was analyzed by STRING software (http://www.string-db.org/).

### Enzyme-Linked Immunosorbent Assay (ELISA) Methods

The human prostatic acid phosphatase (PAP, ACPP) ELISA kit (ab267802; Abcam, Cambridge, MA, USA; detection limit, 9.73 pg/mL; SwissProt: P15309), human CD5 antigen-like (CD5L) ELISA kit (ab213760; Abcam, Cambridge, MA, USA; detection limit, < 10 pg/mL; SwissProt: O43866), human alpha 2 macroglobulin (A2M) ELISA kit (ab108883; Abcam, Cambridge; detection limit, 1.25 μg/mL; SwissProt: P01023), human peptidyl-prolyl cis-trans isomerase A (PPIA) ELISA kit (CSB-E09920h; CUSABIO Co., Wuhan, China; detection limit, 0.78 ng/mL; SwissProt: P62937), and human beta-actin (ACTB) ELISA kit (CSB-E13298h; CUSABIO Co.; detection limit, 0.078 ng/mL; SwissProt: P60709) were used to detect protein levels in follicular fluid. Ovarian follicular fluid samples were diluted 1:100 and 1:100 for ACPP and A2M, respectively. The protein concentrations were measured according to the manufacturer’s instructions.

### Western-Blot Analysis

Each of 10 μg cumulus cells protein samples were subjected to polyacrylamide gel electrophoresis, and were transferred onto a PVDF membrane (IPVH00010, Millipore, Massachusetts, USA). Further, 5% (w/v) skimmed milk was used to block the above membrane at 37°C for 2 h, and the primary antibodies (CD5L, ab45408, Abcam; ACPP, 60176-1-Ig, Proteintech Group, Chicago, USA; β-Actin, 66009-1-Ig, Proteintech Group) were respectively added for incubation at 4°C overnight. Secondary antibodies as goat anti-mouse IgG-HRP (BK0023, BEST, Xian, China) and goat anti-rabbit IgG-HRP (BK0027, BEST) were then incubated with membrane at room temperature for 1.5 h. The blots were visualized using the ECL Plus Luminous Kit (S17851, Yeasen, Shanghai, China). At last, the results were measured with Image J software.

### Statistical Analysis

The parametric data were analyzed using t-tests to compare the means of two groups. Nonparametric analysis was performed using the Mann-Whitney U-test. Parametric data are presented as the mean ± SD, while nonparametric data are presented as the median ± IQR. Receiver operating characteristic (ROC) curves were calculated by using MedCalc Software (Version 12.4.2.0, Belgium). The diagnostic score of MII oocytes and DOR patients was set to 1, whereas that of Gv oocytes and controls was set to 0. The Pearson correlation method was performed to determine the association between two different parameters. An r value within the range of −0.4 to −0.1 or 0.1 to 0.4 indicates a weak correlation, a value within the range of −0.7 to −0.4 or 0.4 to 0.7 indicates a moderate correlation, and a value within the range of −1.0 to −0.7 or 0.7 to 1.0 indicates a strong correlation. *P*-values <0.05 were considered statistically significant by SPSS software (Chicago, IL, version 18.0). Our clinical data provided 85.92% power to identify significant differences between MII and Gv oocytes at a statistical support level of α=0.05 with d = 0.6 applying a one-tail model calculated by Gpower 3.0.5 and provided 75.47% power to identify significant differences between DOR patients and controls with the same parameters.

## Results

### Proteomics Characterization

Three biological replicates per group were included in this analysis according to the PCA results, excluded one sample with large dispersion. We identified a total of 333 proteins through iTRAQ-2D LC-MS/MS. Among the 333 identified proteins, there were 31 differentially expressed proteins in MII oocytes compared with Gv oocytes, including 10 upregulated proteins (>1.50-fold, *P*<0.05) and 21 downregulated proteins (<0.67-fold, *P*<0.05) (**Table 1**).

**Table 1:** Differentially expressed proteins and their expression levels quantified by iTRAQ-2DLC-MS/MS.

Protein ID	Protein names	Gene names	iTRAQ ratio	*P* value
**Increased in MII**				
P59666	Neutrophil defensin 3	DEFA3	2.875	0.010
Q01459	Di-N-acetylchitobiase	CTBS	2.045	0.039
P15309	Prostatic acid phosphatase	ACPP	2.015	0.002
P06858	Lipoprotein lipase	LPL	1.872	0.027
P08253	72 kDa type IV collagenase	MMP2	1.729	0.042
O14646	Chromodomain-helicase-DNA-binding protein 1	CHD1	1.512	0.012
P98066	Tumor necrosis factor-inducible gene 6 protein	TNFAIP6	1.452	0.048
P01344	Insulin-like growth factor II	IGF2	1.291	0.042
D6RFX5	Amphiregulin	AREG	1.240	0.041
A0A087X1J7	Glutathione peroxidase	GPX3	1.225	0.043
**Decreased in MII**				
P62805	Histone H4	H4C1	0.128	<0.001
P02775	Platelet basic protein	PPBP	0.226	0.018
P69905	Hemoglobin subunit alpha	HBA1	0.278	<0.0001
P68871	Hemoglobin subunit beta	HBB	0.311	0.019
P01871	Immunoglobulin heavy constant mu	IGHM	0.345	0.001
P04114	Apolipoprotein B-100	APOB	0.400	<0.0001
P04406	Glyceraldehyde-3-phosphate dehydrogenase	GAPDH	0.486	0.011
Q8N139	ATP-binding cassette sub-family A member 6	ABCA6	0.491	0.012
O43866	CD5 antigen-like	CD5L	0.513	0.001
P02745	Complement C1q subcomponent subunit A	C1QA	0.535	0.002
P62937	Peptidyl-prolyl cis-trans isomerase A	PPIA	0.544	0.022
P01023	Alpha-2-macroglobulin	A2M	0.556	0.010
Q5TZA2	Rootletin	CROCC	0.580	0.021
P04003	C4b-binding protein alpha chain	C4BPA	0.604	0.001
B4DPQ0	Complement C1r subcomponent	C1R	0.645	0.006
P60709	Actin, cytoplasmic 1	ACTB	0.681	0.020
D6R934	Complement C1q subcomponent subunit B	C1QB	0.716	0.023
P02671	Fibrinogen alpha chain	FGA	0.747	0.026
P09871	Complement C1s subcomponent	C1S	0.747	0.019
P02675	Fibrinogen beta chain	FGB	0.775	0.041
A0A0J9YX35	Immunoglobulin heavy variable 3-64D	IGHV3-64D	0.786	0.013

### Bioinformatics Analysis

**Figure 2:**
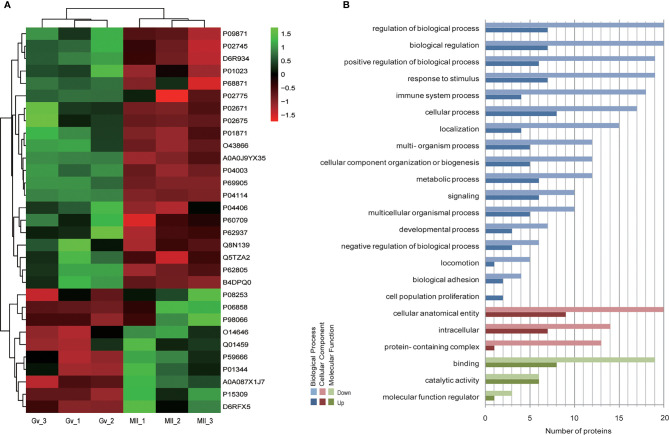
Bioinformatics analysis of differentially expressed follicular fluid protein biomarker candidates for mature oocytes. **(A)** Clustering analysis; **(B)** Gene Ontology (GO) analysis.

Hierarchical clustering provided a visualized mode to display the clustering patterns of the differentially expressed proteins (up and down) between the two groups (**Figure 2A**). GO analysis for differentially expressed proteins revealed that ‘cell population proliferation’ was the most diverse enrichment trend between up- and downregulated proteins (**Figure 2B**).

**Figure 3:**
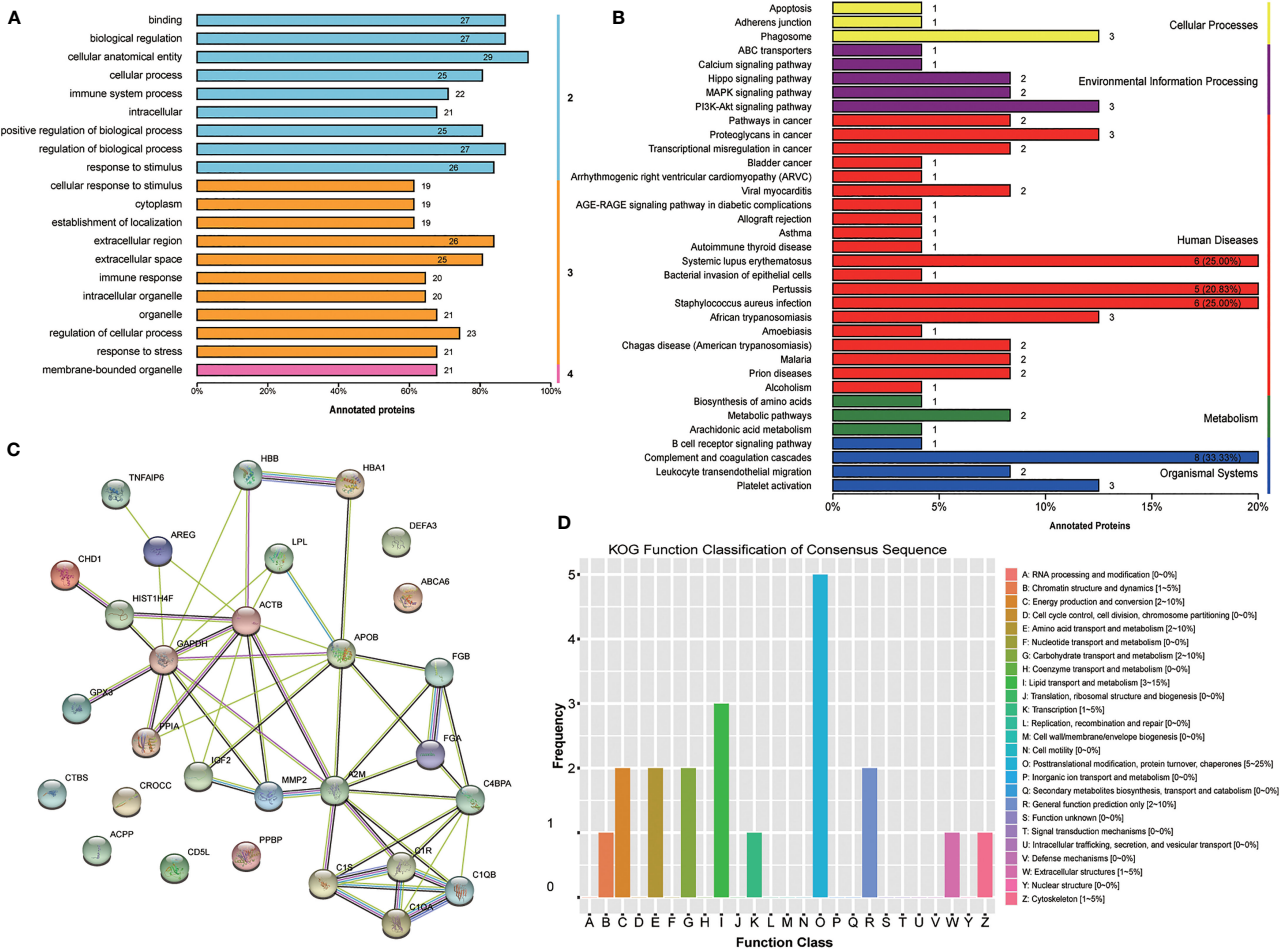
Bioinformatics analysis of the set of follicular fluid protein biomarker candidates for mature oocytes. **(A)** Gene Ontology (GO) level classification; **(B)** Kyoto Encyclopedia of Genes and Genomes (KEGG) enrichment classification; **(C)** STRING analysis; **(D)** Clusters of Protein Homology (KOG) function classification.

Furthermore, GO level classification of differentially expressed proteins revealed that most of the proteins were involved in cellular anatomical entity (29 proteins), extracellular region (26 proteins), and membrane-bounded organelle (21 proteins) (**Figure 3A**). Directed acyclic graphs showed that the most significant GO terms were regulation of cell-cell adhesion, extracellular exosome, and serine-type endopeptidase inhibitor activity for biological process, cellular component, and molecular function, respectively (**Supplementary Figures 1**-**3**). KEGG enrichment classification revealed some significant pathways: phagosome process (3 proteins) and the PI3K-Akt signaling pathway (3 proteins) (**Figure 3B**). In addition, STRING analysis found interactions between these proteins (**Figure 3C**). Finally, we analyzed the COG annotation and classification and found that posttranslational modification, protein turnover, and chaperones (2–25%) might be some important physiological processes involved during the MII stage. KOG analysis confirmed this result and further highlighted lipid transport and metabolic processes (**Figure 3D**).

### Biomarker Validation and ROC Analysis

**Figure 4:**
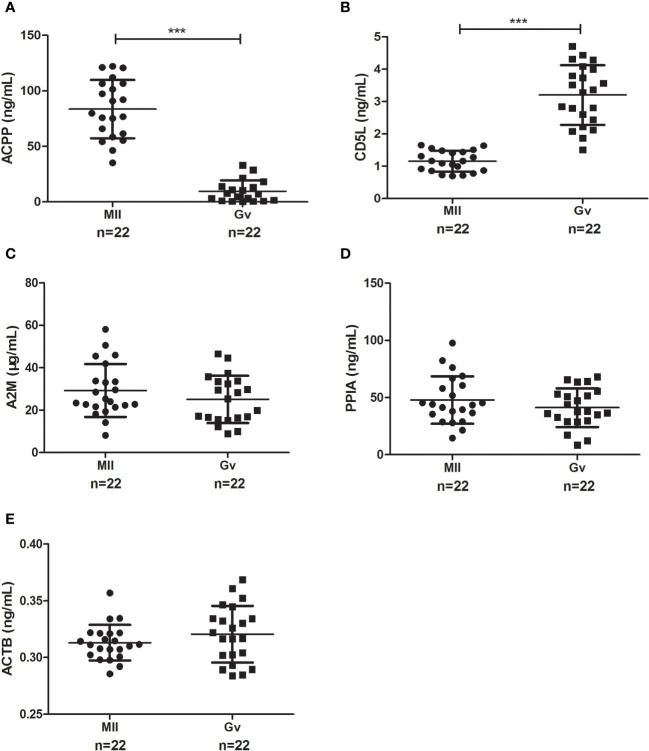
Enzyme-linked immunosorbent assay results of candidate proteins in MII and Gv oocytes in follicular fluid. **(A)** Prostatic acid phosphatase (ACPP); **(B)** CD5 antigen-like (CD5L); **(C)** Alpha 2 macroglobulin (A2M); **(D)** Peptidyl-prolyl cis-trans isomerase A (PPIA); **(E)** Beta-actin (ACTB). ^***^ *P* < 0.0001.

To confirm our proteomics results, we verified the follicular fluid levels of ACPP, CD5L, A2M, and PPIA by ELISA in ICSI patients with both MII and Gv oocytes (n=22). We found a significantly higher level of ACPP (83.56 ± 26.26 vs. 9.56 ± 9.83 ng/mL, *P*<0.0001, **Figure 4A**) and a significantly lower level of CD5L (1.16 ± 0.32 vs. 3.20 ± 0.92 ng/mL, *P*<0.0001, **Figure 4B**) in MII oocyte follicular fluid. Furthermore, no significant differences in A2M, PPIA, or ACTB were found (*P*>0.05, **Figures 4C–E**).

We performed a ROC analysis to evaluate the sensitivity and specificity of the two proteins, and the areas under the curve (AUCs) between MII and Gv oocytes were 1.000 for ACPP and 0.994 for CD5L. The sensitivity values were 100.00% and 100.00% for ACPP and CD5L, respectively, and the specificity values were 100.00% and 95.45%, respectively.

### Further Measurement in DOR Patients

**Figure 5:**
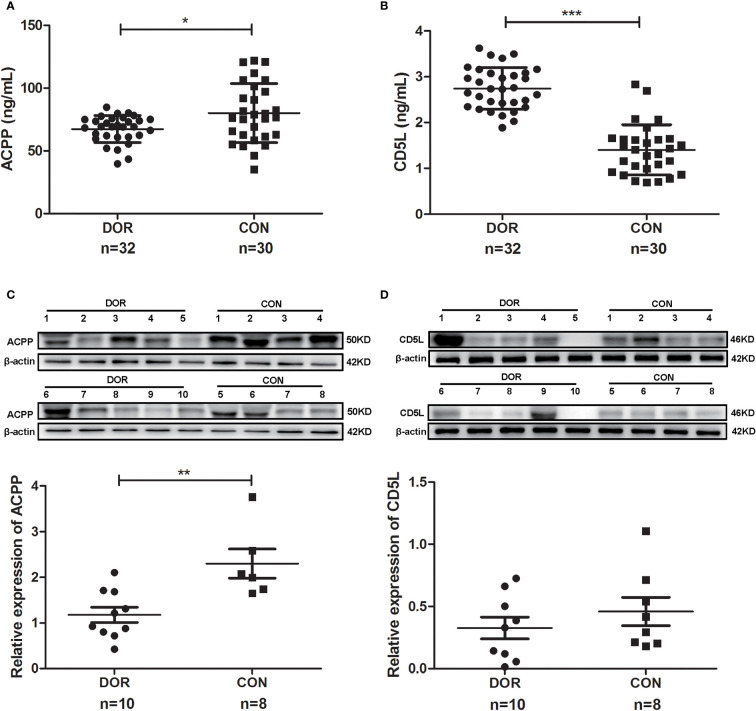
Proteins expression levels change between diminished ovarian response (DOR) patients and controls (CON) in follicular fluid and cumulus cells. **(A)** Prostatic acid phosphatase (ACPP) and **(B)** CD5 antigen-like (CD5L) levels in follicular fluid were analyzed by enzyme-linked immunosorbent assay. **(C)** ACPP and **(D)** CD5L levels in cumulus cells were analyzed by western-blot. ^*^ *P* < 0.05; ^**^ *P* < 0.01; ^***^ *P* < 0.0001.

Further study showed a significantly lower level of follicular fluid ACPP (67.36 ± 10.77 vs. 80.10 ± 23.51 ng/mL, *P*=0.028, **Figure 5A**) in DOR patients (n=32), together with a higher level of CD5L (2.74 ± 0.45 vs. 1.41 ± 0.55 ng/mL, *P*<0.0001, **Figure 5B**). In identifying DOR patients, the AUC, sensitivity and specificity of ACPP were 0.666, 77.42%, and 62.07%, respectively, the AUC, sensitivity and specificity of CD5L were 0.959, 93.75%, and 93.10%, respectively.

Otherwise, ACPP and CD5L levels in cumulus cells were analyzed by western-blot. We found significantly lower level of ACPP (**Figure 5C** and **Supplementary Figure 4**) in DOR patients using grayscale detection by Image J (0.51 fold, *P*=0.004). However, CD5L level downregulated in DOR patients without significant change (**Figure 5D** and **Supplementary Figure 4**).

The clinical data analysis revealed significant differences in the levels of the following between DOR patients and controls (*P*<0.05): AMH, AFC, the number of retrieved oocytes, follicle-stimulating hormone (FSH), estradiol (E2), and luteinizing hormone (**Table 2**). Then, all DOR patients were separated by age, body mass index, AMH, AFC, and other clinical data. In the Pearson correlation analysis, we found significant moderate positive correlations between CD5L and age or FSH (r=0.47, *P*=0.0001; r=0.60, *P*<0.0001, respectively). Additionally, significant strong negative correlations were observed between CD5L and AMH, AFC, and the number of retrieved oocytes (r=-0.73, *P*<0.0001; r=-0.81, *P*<0.0001; r=-0.72, *P*<0.0001, respectively). Furthermore, weak positive correlations were observed between ACPP and the AFC and number of retrieved oocytes (r=0.32, *P*=0.013; r =0.31, *P*=0.016, respectively).

**Table 2:** Characteristics of diminished ovarian response (DOR) patients and controls.

Characteristic	DOR group (n = 32)	Control group (n = 30)
Age (year)^a^	34.56 ± 4.72	32.30 ± 4.15
Infertility years (year)^b^	3.00 ± 3.81	2.00 ± 2.75
BMI (kg/m2)^b^	22.80 ± 4.20	20.70 ± 4.30
AMH (ng/mL)^a^	0.80 ± 0.28	4.53 ± 2.11^***^
AFC^b^	4.00 ± 2.00	14.00 ± 6.00^***^
Number of retrieved oocytes^b^	4.00 ± 2.00	15.00 ± 10.00^***^
Basal hormone level		
Follicle-stimulating hormone (IU/L)^b^	9.36 ± 6.97	4.89 ± 3.00^***^
Estradiol (pg/mL)^b^	44.00 ± 36.00	29.00 ± 26.25^***^
Progesterone (ng/mL)^b^	0.64 ± 0.82	0.55 ± 0.56
Prolactin (ng/mL)^b^	13.49 ± 10.29	13.01 ± 8.73
Luteinizing hormone (IU/L)^b^	3.69 ± 1.59	2.33 ± 1.61^**^
Testosterone (ng/mL)^b^	0.46 ± 0.30	0.48 ± 0.36

Parametric data are presented as the mean ± SD, while nonparametric data are presented as the median ± IQR. BMI, body mass index; AMH, anti-Mullerian hormone; AFC, antral follicle count. ^a^P-value between two groups using the t-test; ^b^P-value between two groups using the Mann-Whitney U-test; ^**^P < 0.01; ^***^P < 0.0001.

## Discussion

Follicular fluid is a plasma transudate that fills the follicle antrum and provides a microenvironment for oocyte maturation. Thus, its composition may have a direct influence on the oocyte, in both its ability to mature and its quality (15). The mechanism by which proteomes affect oocyte maturity and quality remains unclear. To date, several researches focused on the alterations in follicular fluid proteins during folliculogenesis and oocyte maturation have been already published. Ambekar et al. revealed the presence of 480 proteins in follicular fluid during oocyte maturation and ovulation without validation and Controls (16). Lewandowska et al. obtained a list of 20 proteins possibly associated with oocyte maturity, also have not validated their differentially expressed proteins by focusing more on the proteomics method comparison (17). Pla et al. revealed insights in folliculogenesis and oocyte maturation by screening the proteome of small antral follicles containing oocytes capable or not to reach metaphase II during *in vitro* maturation (IVM), giving us more thinking on the extensiveness and continuity of the sample selection in oocyte maturation (8).

In this study, we investigated proteomic expression in ovarian follicular fluid from women with different oocyte maturities by iTRAQ-2D LC-MS/MS and found 31 significantly differentially expressed proteins between the mature and immature groups. ACPP and CD5L were validated in a larger sample as the biomarkers for oocyte maturity; moreover, the expression levels in DOR patients suggested a potential role for ACPP and CD5L in measurement of oocyte quality. However, A2M, PPIA, and ACTB showed no significant differences between MII and Gv oocytes by ELISA validation. This might due to the larger sample size of followed validation reduces the sampling error of iTRAQ-2D LC-MS/MS, which is common in proteomic studies (18, 19).

We screened 31 differentially expressed proteins in MII oocytes compared with Gv oocytes, including 10 upregulated proteins (>1.50-fold, *P*<0.05) and 21 downregulated proteins (<0.67-fold, *P*<0.05), by iTRAQ-2D LC-MS/MS and bioinformatics analysis (**Table 2**). The differentially expressed proteins obtained in this study have also been certified in other proteomics studies. PPIA was also revealed to be downregulated in the final developmental stage of mouse follicles *in vitro* by 1D and 2D LC-MS/MS analysis (20). Amphiregulin (AREG, SwissProt: D6RFX5) and tumour necrosis factor-inducible gene 6 protein (TNFAIP6, SwissProt: P98066) are two unregulated proteins found in human follicular fluid after induction of ovulation by LC-MS/MS (21). Our results contain known proteins associated with oocyte development, giving us more confidence on our proteomics results. Furthermore, some proteins we screened were already reported to be related to folliculogenesis, oocyte maturation, and ovulation. AREG is a member of the epidermal growth factor (EGF) family, which can transduce luteinizing hormone (LH) signals from granulosa cells to oocytes, resulting in meiosis resumption (22). TNFAIP6 cooperates with inter-alpha inhibitors and pentraxin 3 to ensure balanced cumulus expansion (23), mediating the release of oocytes. Glutathione peroxidase (GPX3, SwissProt: A0A087X1J7) might peak to suppress the oxidative stress resulting from the accumulation of reactive oxygen species (ROS) during IVM (24).

KEGG analysis showed that phagosome, the PI3K-Akt signaling pathway, the MAPK signaling pathway, and the Hippo signaling pathway were among the most enriched processes (**Figure 3B**). MAPK activities regulated by GnRH are necessary for normal fertility (25), while the PI3K pathway has been reported to be a key pathway in follicle activation and growth, precisely in gonadotropin stimulation of meiotic resumption (26, 27). Most follicular fluid exudates are contributed by cumulus cells, oocytes and granulosa cells at large during oocyte maturation; so we took our results as a reflection of the microenvironment influenced by inner cells. Various paracrine/autocrine factors were reported as controller in folliculogenesis. Considering this, we prefer to study follicular fluid and cumulus cells (also granulosa cells, cumulus-oocyte complexes) together to complement each other.

A higher level of ACPP in follicular fluid was associated with increased oocyte cleavage (28) and ovulation (21) in prior studies. In our study, consistent results showed higher levels of ACPP in mature oocytes. Lysophosphatidic acid (LPA) has been recognized as an enhancer in human oocyte maturation *in vitro* (29) and can be inactivated by ACPP (30). We hypothesized that in matured MII oocytes, rising levels of ACPP in follicular fluid may act to remove an excessive maturational effect of LPA. Consequently, the lower level of ACPP in DOR patients might also related to the level of LPA. Together, the level of ACPP in follicular fluid can be used as a potential marker for predicting oocyte maturation and developmental potential.

CD5L is a secreted glycoprotein by macrophages and circulates in the blood, also known as Sp alpha and apoptosis inhibitor of macrophages, and transported in the cytoplasm *via* CD36-mediated endocytosis (31). In follicular granulosa cells, Osz et al. found that CD36 inhibition increased cell proliferation and decreased apoptosis (32), while Wu et al. found that CD36 overexpression inhibited cell proliferation and promoted cell apoptosis (33). In addition, CD5L induces autophagy through the PI3K-Akt signaling pathway by interacting with CD36 on the cell surface to promote antiapoptotic outcomes (34). Considering our results, endocytosis for CD5L to play antiapoptotic effect might not the most important reason for the lower level of CD5L in control subjects. However, the specific mechanism needs further study.

Composition in follicular fluid may have a direct influence on the oocyte maturation and its quality. And, the lower apoptotic rate in cumulus cells might be an indicator of good oocyte quality, in terms of a greater capacity in oocyte developmental potential (35). Molecules screened by omics, especially with antiapoptotic effects might be potential supplements improving IVM, which need further study.

## Conclusions

In summary, our study investigated the changes in proteomics affecting oocyte maturity in human ovarian follicular fluid, providing possible biomarkers to determine oocyte maturity. Our study also paves the way for further investigation of the role of ACPP and CD5L in maintaining oocyte quality in the ovarian microenvironment.

## Data Availability Statement

The datasets presented in this study can be found in online repositories. The names of the repository/repositories and accession number(s) can be found below: http://www.proteomexchange.org/, PXD027577.

## Ethics Statement

The studies involving human participants were reviewed and approved by Ethics Committee of the Faculty of Hangzhou First People’s Hospital. The patients/participants provided their written informed consent to participate in this study.

## Author Contributions

CW performed the experiments and wrote the paper. XF and YF conceived and designed the experiments. HZ and WZ collected the clinical data. ZC. and YF analyzed the data and prepared the figures and tables. All authors reviewed and approved the final version of the manuscript.

## Funding

This research was supported by the Zhejiang Provincial Natural Science Foundation of China (Grant No. LQ18H040009).

## Conflict of Interest

The authors declare that the research was conducted in the absence of any commercial or financial relationships that could be construed as a potential conflict of interest.

## Publisher’s Note

All claims expressed in this article are solely those of the authors and do not necessarily represent those of their affiliated organizations, or those of the publisher, the editors and the reviewers. Any product that may be evaluated in this article, or claim that may be made by its manufacturer, is not guaranteed or endorsed by the publisher.
