# Supplementary figures and images for: Proteomic Analysis of the Alterations in Follicular Fluid Proteins During Oocyte Maturation in Humans
Source: Front Endocrinol (Lausanne). 2022 Feb 3;12:830691. doi: 10.3389/fendo.2021.830691 (PMC8850365; doi:10.3389/fendo.2021.830691)

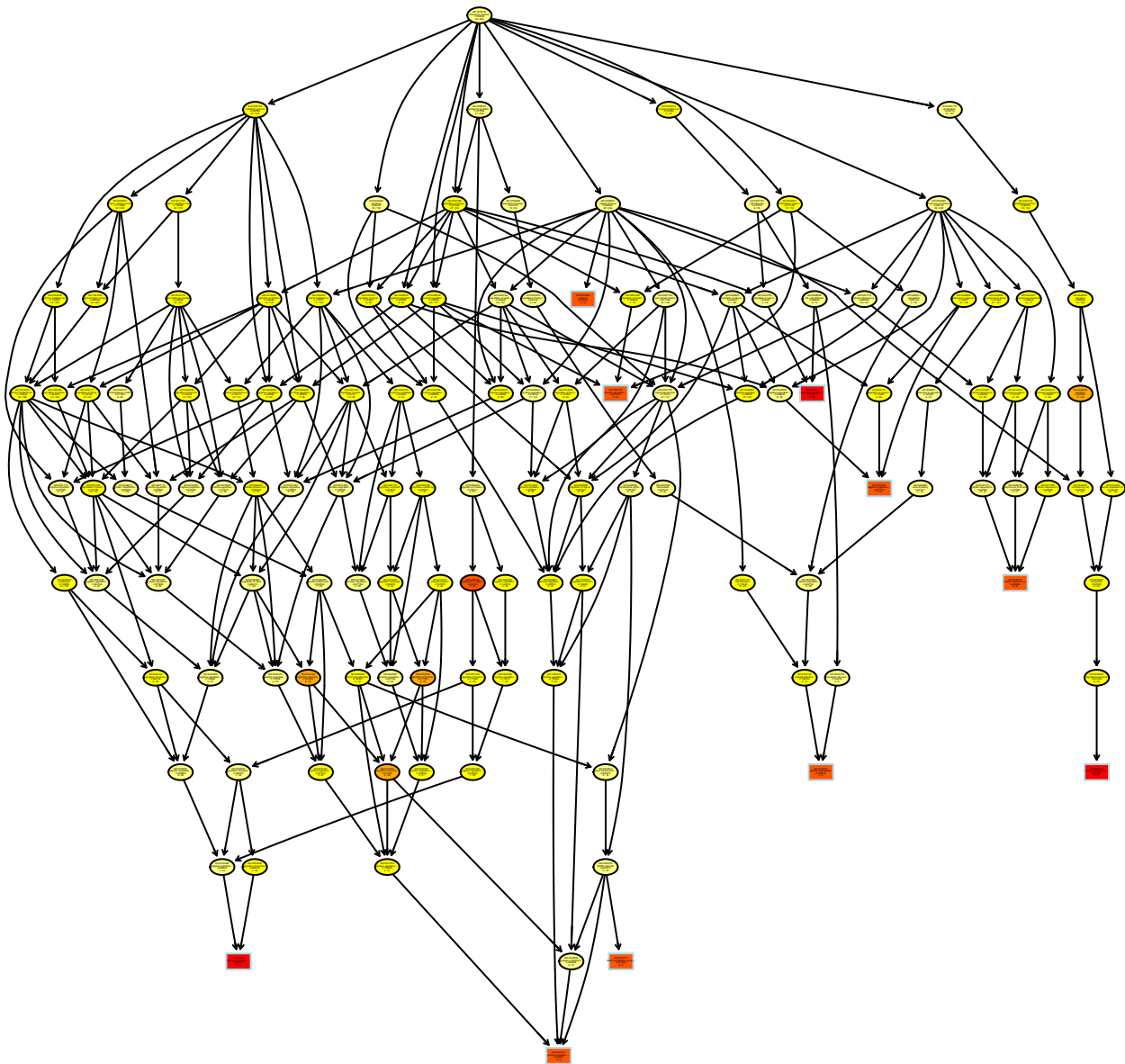

Supplement: Supplementary file 1 [file DataSheet_1.pdf]

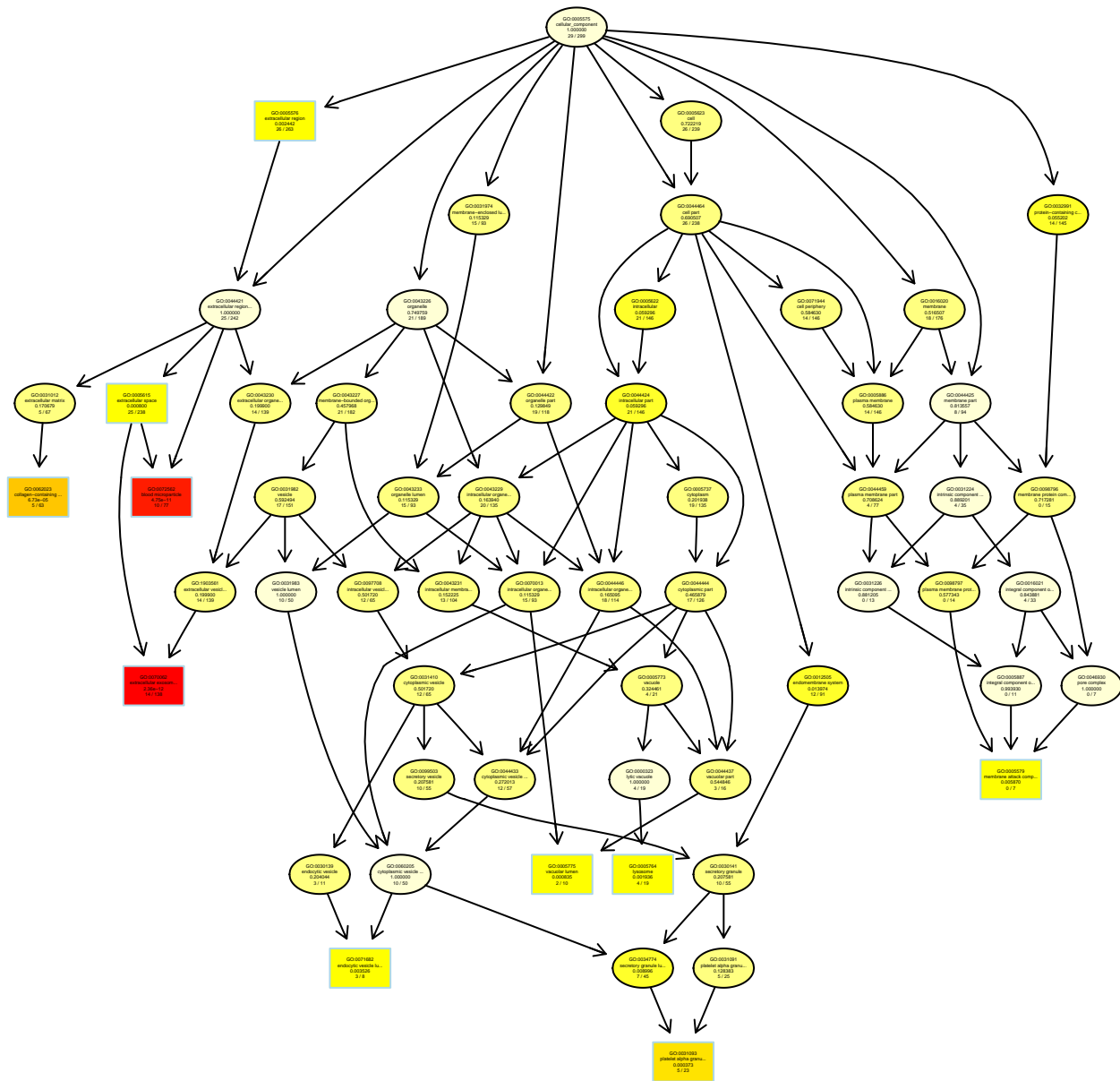

Supplement: Supplementary file 2 [file DataSheet_2.pdf]

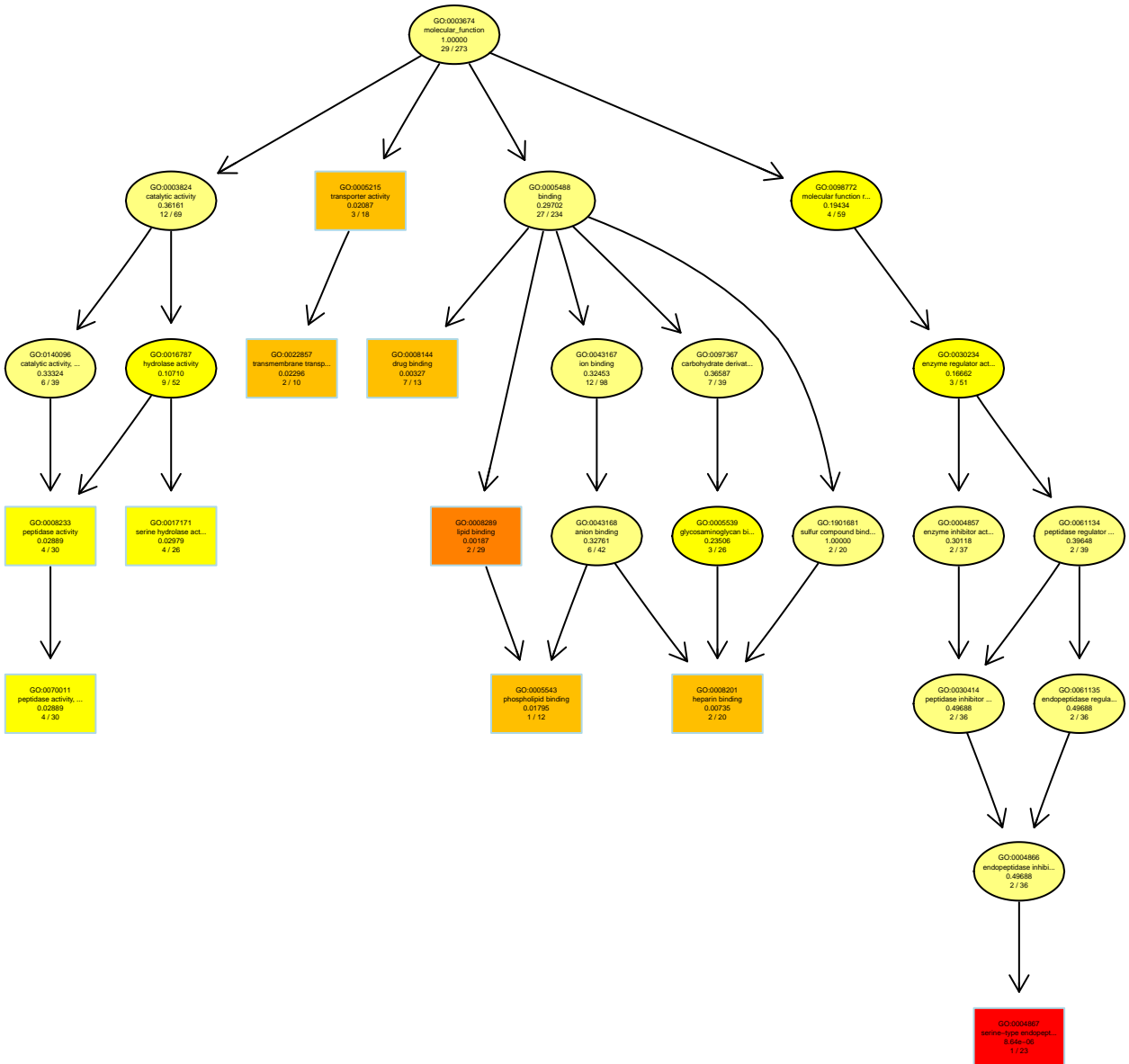

Supplement: Supplementary file 3 [file DataSheet_3.pdf]

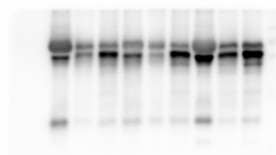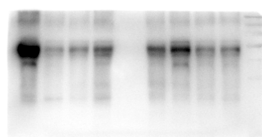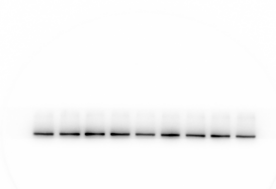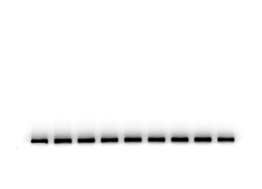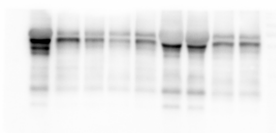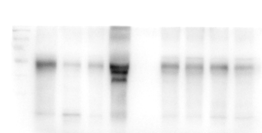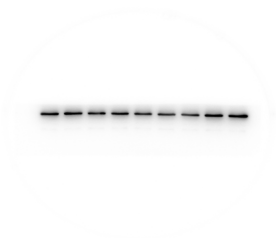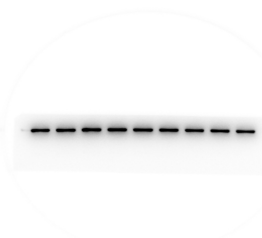

Supplement: Supplementary file 4 [file DataSheet_4.pdf]
